# Mr. Hill's Essay on the Nature and Cure of Insanity

**Published:** 1816-03

**Authors:** 


### *Mr. Hill's Essay on the Nature and Cure of Insanity.*

( Concluded from p. 129. )

Our author divides the *exciting* causes of insanity into two distinct classes, the conjunction of which forms also a third. The first, or causes *ab externo,* act on the system through the medium of the passions, " or mechanical violence acting on predisposition." The second are, " those which originate directly from the lesion of some organic function acting upon predisposition." The third is formed from a conjunct action of the two former. Ferguson, the Scotch poet, is adduced as an illustration of this statement. Juvenile indiscretions founded the predisposition to insanity, which was called into action by a fall from a stair-case. The horror of a lunatic asylum completed the catastrophe.

We find it so absolutely impossible to connect in the way of analysis, our author's chain of reasoning on any subject, that we are forced on this occasion to deviate from our usual course, and select such passages from the different Sections of the work as may appear most useful to the reader, and creditable to the author.

" Early imprudent use of alcohol, it is much to be feared, has often arisen from the careless or injudicious advice of the faculty, which circumstance, added to the immense use of Charlatanic compositions, prove but too successful causes exciting to lunacy, suicide, and *honorable* or *dishonorable* murder. Many persons who despise the degrading epithet of drunkard, are yet daily or nightly accustomed to such spirituous potations as scarce ever to retire to rest without verging to this state. Defect, or want of long accustomed impressions, are the origin of morbid perceptions and erroneous judgment, as the affluent suddenly becoming poor, the poor suffering from famine, both losing the influence of impressions necessary to regular and sane perceptions. Men on acquiring riches retire from active life, change the sources of their impressions, rush into the country, embrace dulness, feel *ennui,* hypochondriacal horrors, fall into insanity, and commit suicide. This climax is completed, not by pure mental exciting causes, but arises from corporeal changes, such as from a life of activity, gentle regular exercise, temperance, and good general health, to one of idleness, full living, and dissipation; hence, visceral obstructions, &c."

"The exciting causes of insanity, which may be denominated internal, are nearly as numerous as those which have been considered as more remote or external. The current of common opinion has been in favour of the brain being always the seat of internal causes, or at least, when the disease appears in the high form. A question has also arisen respecting this organ after dissection. *Indiscriminè utrum.*"

Are the organic changes of the brain discovered after death, the causes or the effects of the disease? No dependance can be placed on these appearances, nor on the relations afforded by great anatomists; no useful deductions can be made from their nicest scrutinies; even the accurate Morgagni affords little upon which reliance can be founded. Lunatics have expired, suffering under extreme violent symptoms connected with both forms, where apparently the most evident brainular affection existed, yet the most able hand and keenest eye have made no correspondent demonstrations. And, *è contra,* considerable local mischief with mental disorder have existed where no approach to actual insanity was ever indicated, even suppuration of the brain, as detected by Dr. Home.

"Thus is deduced decisive evidence, that a certain general state of body is in all cases necessary to the production of insanity; consequently, that brainular derangement may be the leading general exciting cause, and sometimes the particular effect of general leading causes."

The remainder of our extracts shall be from the Appendix of Cases, as containing the most valuable part of the work.

"Case 1.—A youth, æt. 19, marched with his regiment on an urgent expedition across the kingdom, proceeding almost day and night, exposed to excessive fatigue combined with unaccustomed mode of living and defective rest. Upon approaching this city, in the dog-days of 1796, he was seized with a high degree of sthenic insanity, ran across the country, sword in hand, alarming the whole vicinage, fury and horror depicted on his countenance; he was secured and copiously bled by a person unaccustomed to this branch of practice; no benefit arising from this evacuation, it was repeated and some drastic purges administered; but escaping from the house, he rambled ten miles to Chester, was retaken, placed in a dark room on a cool mattress properly secured, and compelled to take a course of cærulean emetics. The stomach resisted the action of six powders of five grains, giving one every half-hour; the seventh was followed by a very smart operation, much tenacious matter was evacuated with bilious coloured dregs. His violence abated. When alone, he was perceived to be constantly moaning with a peculiar inward heavy groan, beating his breast at intervals, but desisting upon any person approaching. It was soon discovered, that he laboured under a syphilitic affection; but the progress of this mischief being stationary, no mercury was given. He was treated for his insanity, conformable to the rules already laid down, viz. *Emetics* three times a week; *camphorated saline mixture*, *mild aperients*, cold shower bath, *digitalis*, dry sparing poor diet, occasional cold spring water turban, and dark room; *camphorated antimonial liniment*, and moderate labour. For the first ten weeks, all his food and medicines were of necessity administered with the iron boat. By unremitted perseverance for four months, all the unfavourable symptoms gave way, followed by lucid intervals and convalescence. This case is inserted to illustrate the fact of the suspension of one species of morbid action during the existence of another. When he came under regular treatment, a chancre was discovered on the prepuce, affording a small quantity of a greenish discharge; this remained nearly *in statu quo* during four months; soon after his first lucid interval it became irritable, and discharged in the usual way, increasing as convalescence advanced. One of the first rational allusions he made was to his misfortune by broken hints whenever I visited him; considerable irritation arose from mercurial friction, with some return of pain in his breast and splenic region."

"Case 3.—J. R. æt. 28, an active farmer, thin and small, but capable of hard labour, having met with a disappointment in the execution of a contract, became drunken, which excess had for some time no other consequences than common. At length, during sober intervals, he began to complain of distressing pain at his breast, accompanied or rather followed by pain across the forehead with confusion of thought, to relieve which, he drank brandy. The mental derangement seemed to commence with the disappointment, and to increase after every act of inebriety, which now had but few intervals. After a long drinking bout, as he termed it, he became furiously maniacal. On my arrival at his house, I found him tied with a cart rope to an upright piece of timber which supported the flooring over the kitchen, frothing at the mouth, swearing horribly, and gesticulating with ungovernable fury. A cærulean *emetic* of seven grains was given him; six such doses, in as many successionary half hours, reduced him to such a state as rendered him fit to be conveyed to a bedstead with a hard smooth mattrass in an adjoining darkened room. The waistcoat being braced on, and his head shaven, a turban imbued with cold spring water, produced a short but refreshing slumber; he awoke with abated turbulence. The *saline acidulated camphor* mixture was now diligently administered in full doses, occasionally interrupted by mild aperients and a dry *emetic* three times a week. His urine was a remarkably deep orange colour, void of sediment, and retained a long time until means were used for its expulsion. *Digitalis*, *antimony*, and *camphor*, combined, formed the evening anodyne, producing good effects. A gradual reduction and final disuse of diffusible stimulus was accomplished; he became reasonable in about eight weeks, described his misery, as he emphatically termed it, as commencing always at his breast, and shooting from thence into his head, when he felt as if scorching up with flames, and as though fire pursued him every where; hence arose an insatiable thirst, accompanied by an ungovernable desire to run away from the torment. He spoke in high terms of the *camphor* mixture, which he said always relieved him, infusing a cool refreshing sensation over the whole system. He continued its occasional use for some time after being capable of exercising his business; but in two years returned to excessive drinking, and again felt peculiar pains, followed by insanity, which was again removed by a recurrence to the same means. After this relapse he remained well seven years, in defiance of excessive bibulation; nor had he a third attack, until the peculiar pain in the breast returned; he now resembled a repeated syphilitic, being with more difficulty restored. This case is sufficient to point out the general run of inebriate cases of lunacy."

"5.—J. B. æt. 27, a London tradesman, becoming deranged, was by accident brought under my care in strange lodgings; he had the most phthisical appearance, was also naturally of a delicate complexion, having large protruding blue eyes, a generally melancholy countenance, often a hectic flush; very irregular as to the alvine and urinary discharges; he had a dry cough; cold clammy morning sweats after watchful moaning nights; pulse on rising, 100 per minute, feeble, but prone to great fluctuation. Dyspepsia to a high degree; yet, from frequent inverted action, great voracity always present, he never completed a meal before *ruminatio* commenced, and it seemed to be nearly all returned by mouthfuls at short intervals, so that in an hour he was ready for a new quantity. He had little or no sickness; but when the first mouthful had returned, the rest was sure to follow; hence the emaciations seemed explained. By the usual methods of observation it was soon discovered, that he had considerable pain in his head, and that he practised manustupration; the use of the waistcoat nearly prevented this practice, but not entirely; for, by assuming certain positions, the wretched effect was often obtained. His mental state corresponded with the corporeal; silly, imbecile, delirious, and when contradicted, very abusive; after a little exertion sinking into stupor, resisting every kind or salutary intention with sullen obstinacy; mischievous and cruel when opportunity occurred, attempting suicide, and then shewing signs of a fear of death. All the means serviceable in melancholia were used for three months with perceptible benefit. One day in my presence, he complained of vertigo and pain in his temples; he had scarce done so a moment, but he sunk from his chair on the floor in a paroxysm of epilepsy; it was severe and long, returning next day with undiminished force; after suffering these attacks for a fortnight with intervals of sense, he died. Two days after, I opened his head. The brain had no preternatural appearances, excepting its ventricles containing about double the usual quantity of fluid; the blood-vessels were pale and empty. A small projection from the inferior anterior edge of the left parietal bone, pointed out the seat of an injury received by a fall from a horse when a boy. The stomach was remarkably smooth on its inner surface, and very thin; the liver small, pale, and tuberculous; mesenteric glands, spleen, and pancreas, enlarged, having a pappy feel. *Vesiculæ seminales* shrunk and empty, though large in capacity; every where evidences of great debility."

The pressure of other works for Review, and the utter impossibility of giving a satisfactory analysis of Mr. Hill's Essay, compel us to desist. We cannot take leave of our author, however, without expressing a sincere esteem for the talents which he unquestionably possesses; and we venture to predict, that they will one day be shewn to greater advantage than in the volume before us. The display of *quotations* invariably disgusts; if they overpower and silence us, they rarely convince. Sound facts, solid inductions, and original trains of thought, are the steps by which an author must now hope to mount the hill of fame. *Learning* is no longer a merit, it is so generally diffused: *Genius* alone can aim at pre-eminence in medicine; and it must be accompanied by indefatigable perseverance, accurate observation, and genuine good sense.

---

